# Anterior Mediastinal Germ Cell Tumor Presenting With Superior Vena Cava Obstruction and Malignant Pericardial Effusion

**DOI:** 10.7759/cureus.113202

**Published:** 2026-07-22

**Authors:** Hamoud Y Obied, Naser Alsharif, Ehab M Ahmed, Abdulhakim Noman, Mohsen A Almahaid, Fahad S Alkusataban, Tehreemah Raziq

**Affiliations:** 1 Surgery, Faculty of Medicine, Najran University, Najran, SAU; 2 Cardiac Surgery, King Khalid Hospital, Najran, SAU; 3 Thoracic Surgery, King Khalid Hospital, Najran, SAU; 4 Cardiology, King Khalid Hospital, Najran, SAU; 5 Cardiac Center, King Khalid Hospital, Najran, SAU; 6 College of Medicine, Alfaisal University, Riyadh, SAU

**Keywords:** anterior mediastinal mass, cardiothoracic surgery, germ cell tumor, pericardial effusion, ptfe graft, superior vena cava syndrome

## Abstract

Primary mediastinal germ cell tumors (PMGCTs) are rare extragonadal neoplasms that most commonly affect young men and may present with compressive cardiopulmonary complications due to their location in the anterior mediastinum. Superior vena cava obstruction and malignant pericardial effusion are serious manifestations that require prompt recognition and coordinated multidisciplinary management. We report a case of a 30-year-old male patient with a large anterior mediastinal germ cell tumor causing superior vena cava (SVC) obstruction and malignant pericardial effusion with impending cardiac tamponade. The patient underwent chemotherapy followed by successful surgical resection with SVC reconstruction using a polytetrafluoroethylene (PTFE) graft under cardiopulmonary bypass. This case underscores the diagnostic complexity of mediastinal germ cell tumors and highlights the importance of multidisciplinary care when these tumors invade or compress great vessels and adjacent cardiac structures.

## Introduction

Primary mediastinal germ cell tumors (PMGCTs) are a rare subset of extragonadal germ cell tumors, accounting for approximately 1-3% of cases, and are predominantly located in the anterior mediastinum [1]. Most cases occur in young adult males between 20 and 35 years of age and may present incidentally or with symptoms related to mass effect, including chest pain, cough, and dyspnea [1,2]. The median tumor size at diagnosis is approximately 9-12 cm [3,4]. In retrospective series, superior vena cava (SVC) obstruction has been observed in 20-50% of patients, while pericardial effusion progressing to tamponade is less common, with an incidence of approximately 6-7% [2].

There are three main categories of PMGCTs: teratomas, seminomas, and nonseminomatous germ cell tumors (NSGCTs). The International Germ Cell Cancer Collaborative Group (IGCCCG) classifies primary mediastinal NSGCTs as poor-risk tumors. NSGCTs are often associated with elevated alpha-fetoprotein (AFP), beta-human chorionic gonadotropin (β-hCG), and lactate dehydrogenase (LDH), which can assist with diagnosis and follow-up. In contrast to gonadal germ cell tumors, PMGCTs do not have well-established environmental or lifestyle risk factors. However, nonseminomatous tumors have been associated with Klinefelter syndrome and hematologic malignancies, including acute leukemias [5].

Due to the rarity of PMGCTs and the absence of formal consensus guidelines, management is generally based on expert opinion, tumor type, disease extent, and patient presentation. Multidisciplinary treatment with neoadjuvant platinum-based chemotherapy is commonly used, followed by surgical resection or radiotherapy to eliminate residual disease [5,6]. In NSGCTs, median sternotomy or thoracotomy may be used to achieve complete resection when feasible. Despite extensive multimodal therapy, nonseminomatous tumors are associated with a poor prognosis, with a five-year overall survival of approximately 40-50% and most relapses occurring within two years of initial treatment [2,5,7]. In this report, we present the case of a 30-year-old male patient with a large anterior mediastinal germ cell tumor complicated by SVC obstruction and malignant pericardial effusion with impending cardiac tamponade, requiring neoadjuvant chemotherapy followed by complex surgical resection and SVC reconstruction.

Germ cell tumors are neoplasms that arise from primordial germ cells, which normally give rise to sperm or ova. They most commonly originate in the gonads, particularly the testes in males, but may also occur in extragonadal locations, such as the mediastinum, retroperitoneum, pineal gland, or sacrococcygeal region. PMGCTs are considered extragonadal germ cell tumors and are thought to result from abnormal migration of germ cells during embryologic development. These tumors should be distinguished from metastatic germ cell tumors, in which a primary gonadal tumor spreads to the mediastinum or other distant sites. Therefore, evaluation for a testicular or other primary gonadal lesion is important before diagnosing a PMGCT. Nonseminomatous mediastinal germ cell tumors are typically aggressive, often present with large anterior mediastinal masses, and may be associated with elevated serum tumor markers such as AFP, β-hCG, and LDH.

## Case presentation

A 30-year-old medically free male patient presented to the emergency department with a one-week history of cough and two weeks of productive sputum. He was afebrile and hemodynamically stable but exhibited prominent neck and forehead vein distension. The patient was alert and oriented (Glasgow Coma Scale (GCS) 15/15) with no neurological deficit or cyanosis. He reported mild weight loss and intermittent low-grade fever. Physical examination showed no lymphadenopathy or peripheral edema.

**Figure 1:**
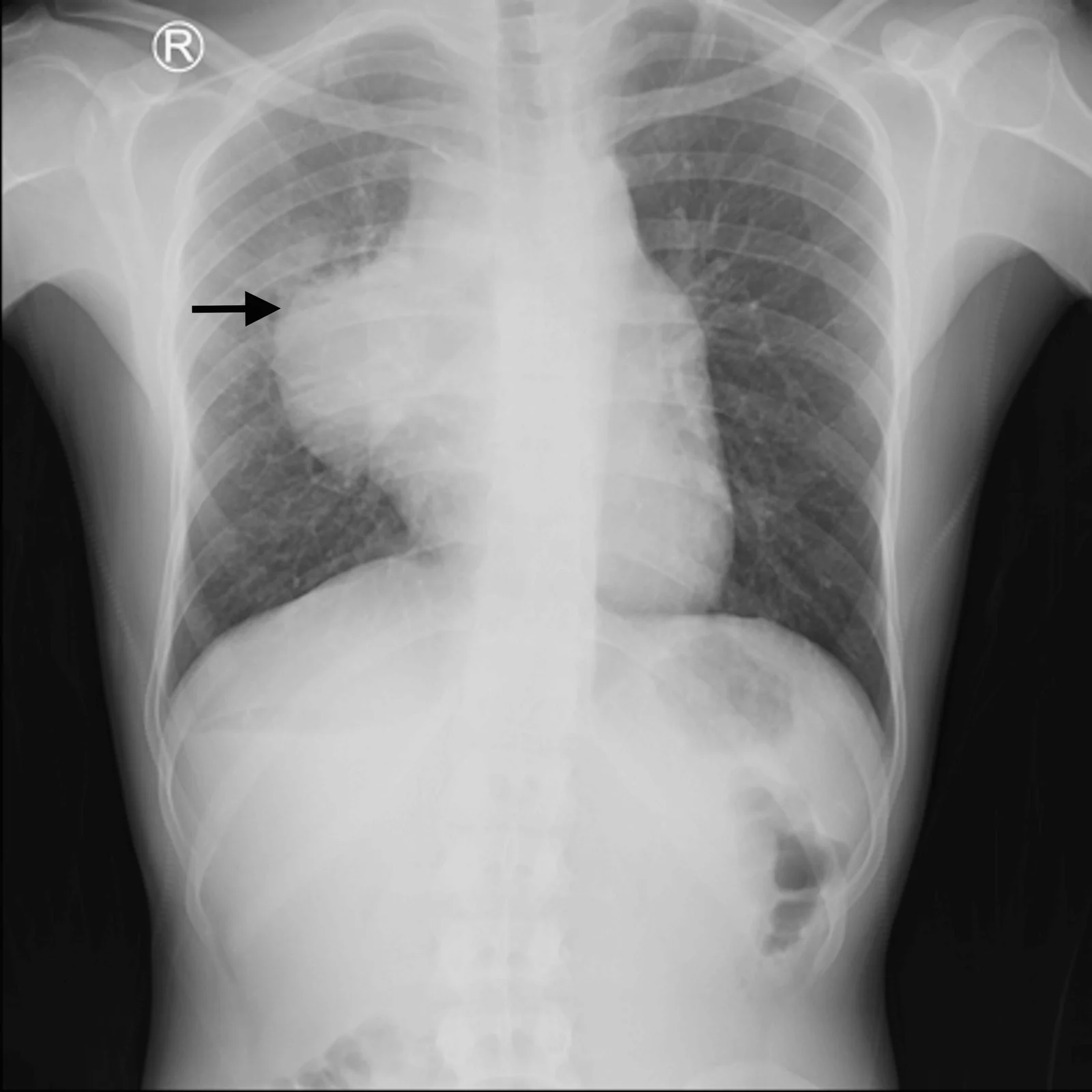
Chest X-ray showing right-sided mediastinal opacity mass (arrow)

**Figure 2:**
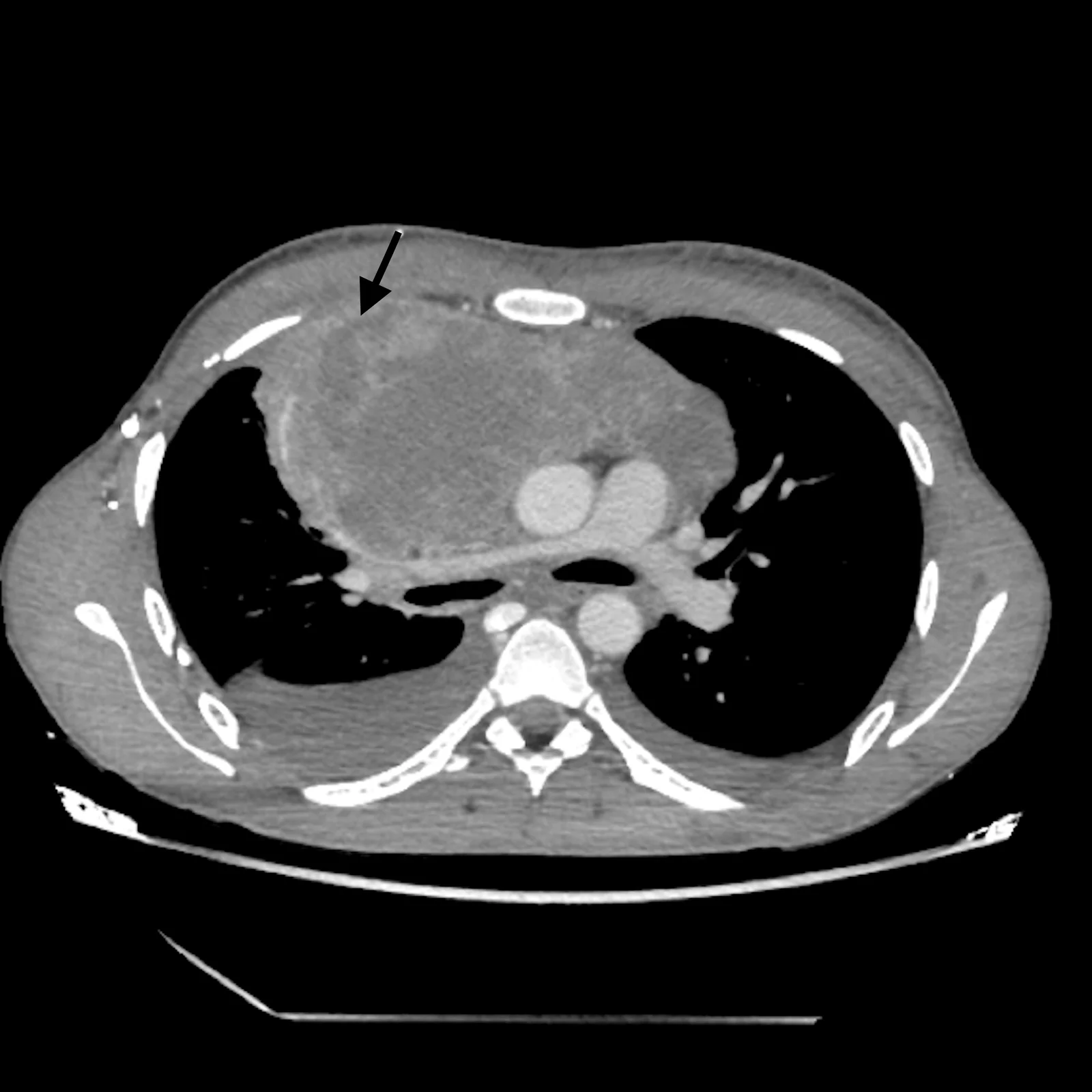
Contrast-enhanced CT chest showing anterior mediastinal mass encasing SVC (arrow) CT: computed tomography; SVC: superior vena cava

Chest X-ray revealed a large right-sided mediastinal mass (Figure 1). Contrast-enhanced CT chest demonstrated a lobulated, heterogeneously enhancing anterior mediastinal mass measuring 8.6 × 15.8 × 8.7 cm, encasing and compressing the SVC with complete occlusion (Figure 2). The mass also compressed the right pulmonary artery and veins and was inseparable from the pericardium. Mild bilateral pleural effusions (right greater than left) and a small pericardial effusion were noted.

Histopathology from ultrasound-guided biopsy revealed necro-inflammatory tissue with atypical cells showing nuclear anaplasia. Immunohistochemistry was positive for glypican-3, AFP, and cluster of differentiation 117 (CD117), and negative for leukocyte common antigen (LCA), cluster of differentiation 30 (CD30), cluster of differentiation 16 (CD16), and placental alkaline phosphatase (PLAP). Tumor markers showed AFP of 686.87 ng/mL (elevated) and β-hCG of 0.93 IU/L (normal). Therefore, the diagnosis of NSGCT was made.

Follow-up echocardiography demonstrated pericardial effusion (2.5 cm behind the right atrium) with impending tamponade. Consequently, pericardiocentesis drained malignant fluid, and the patient received six months of platinum-based chemotherapy. The patient received combination chemotherapy consisting of oxaliplatin 150 mg intravenously, paclitaxel 120 mg intravenously, and gemcitabine hydrochloride 1200 mg intravenously, each administered once during the treatment cycle. Supportive treatment included filgrastim 300 mcg subcutaneously once daily for three days, in addition to antiemetic and hypersensitivity prophylaxis, with marked reduction in tumor size. However, SVC occlusion persisted.

**Figure 3:**
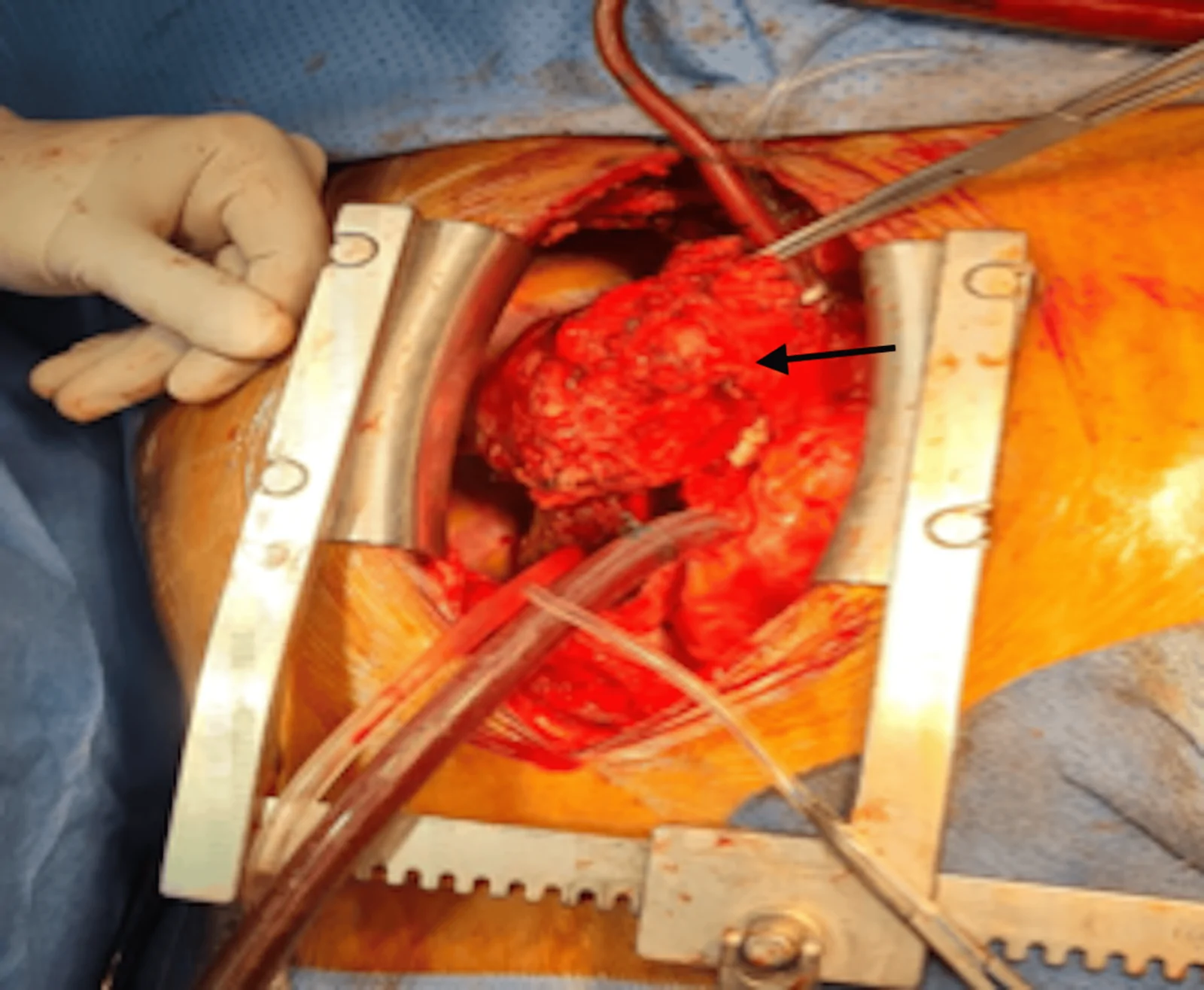
Intraoperative image showing the anterior mediastinal mass. The arrow indicates the region of tumor involvement within the operative field

**Figure 4:**
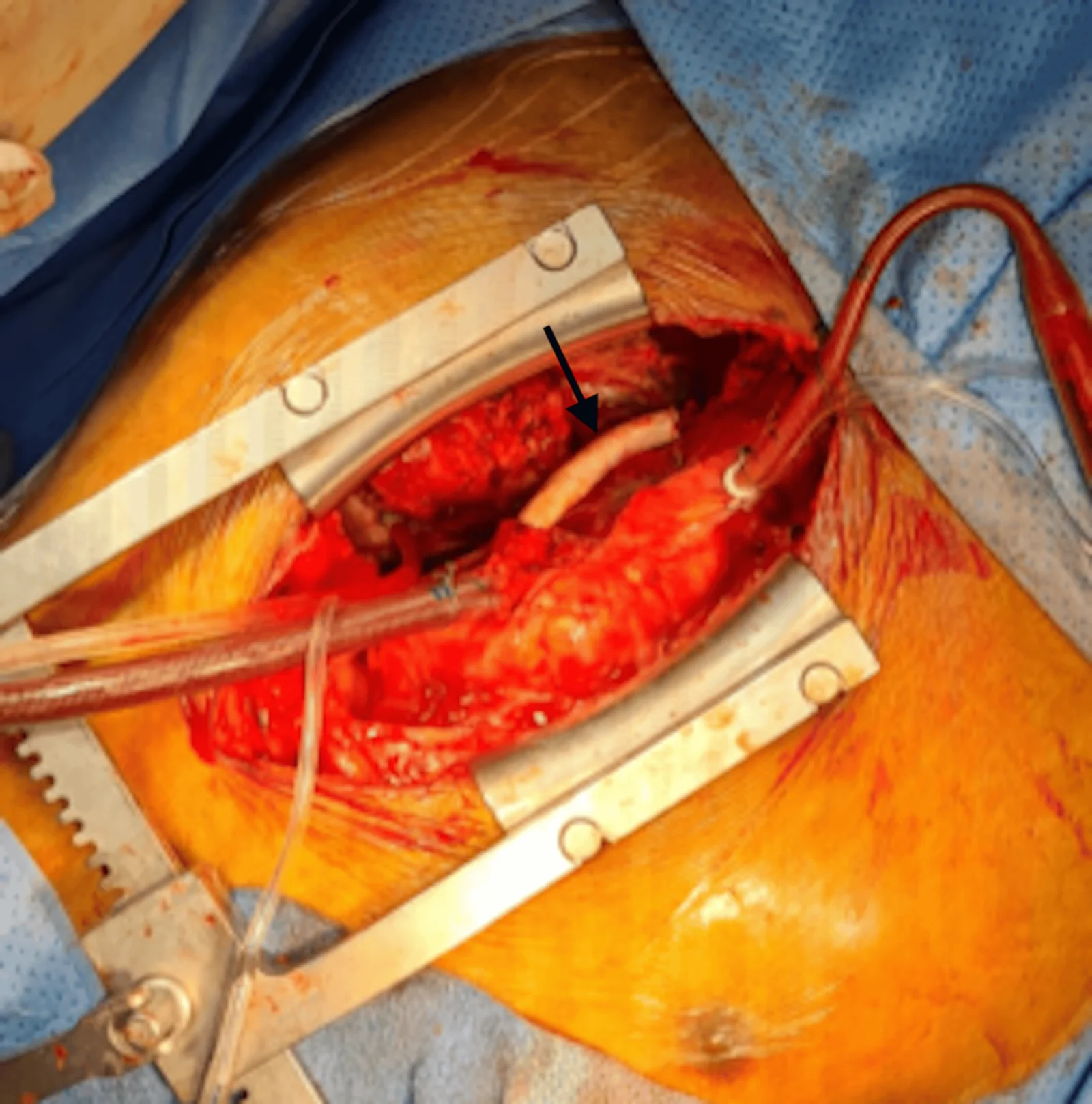
Intraoperative image showing SVC reconstruction with a PTFE graft. The arrow indicates the reconstructed SVC/PTFE graft segment SVC: superior vena cava; PTFE: polytetrafluoroethylene

**Figure 5:**
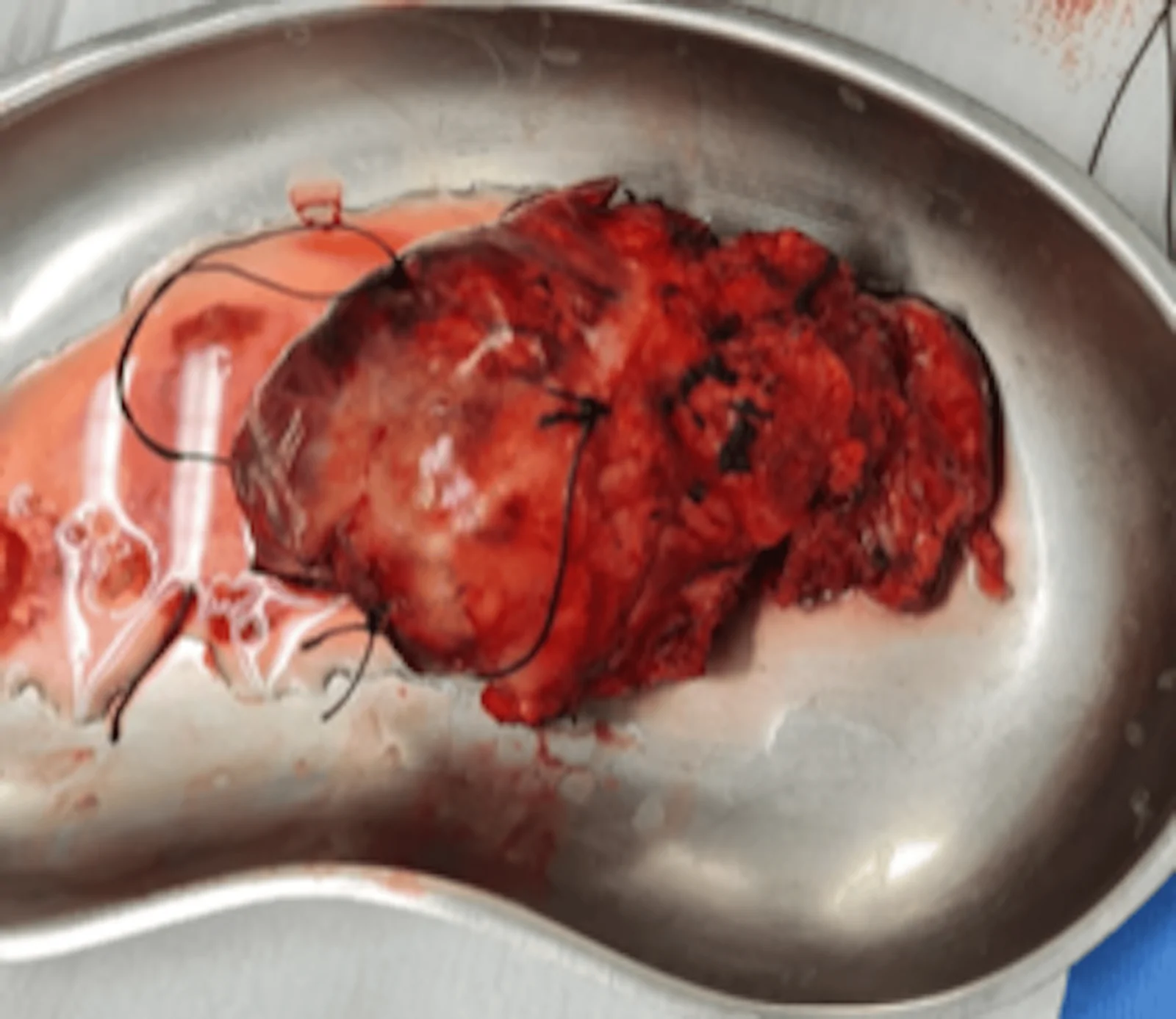
Intraoperative image showing the excised mediastinal mass

A multidisciplinary meeting recommended surgical resection with vascular reconstruction. Median sternotomy was performed under general anesthesia, and the mediastinal mass was carefully dissected (Figure 3). The mass was found to invade the SVC; therefore, segmental SVC resection followed by PTFE graft anastomosis was completed under cardiopulmonary bypass (Figure 4). Adhesiolysis of the pericardium and right lung was performed, and the specimen was excised using an Endo GIA stapler (Medtronic; Minneapolis, Minnesota, USA) (Figure 5).

**Figure 6:**
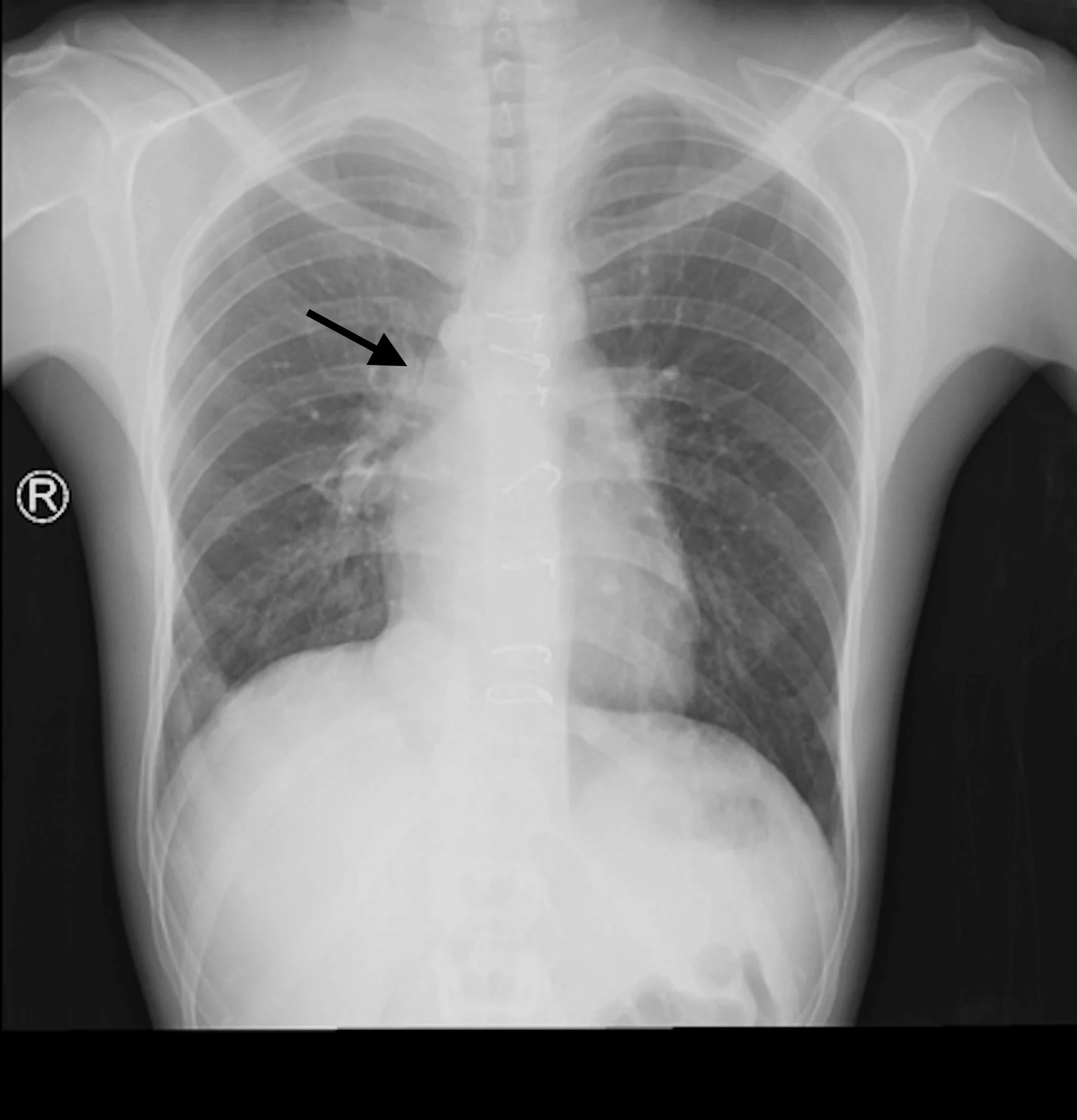
Postoperative chest x-ray showing clear lung fields and absence of the previously seen mediastinal mass. The arrow indicates the postoperative region previously occupied by the mass

**Figure 7:**
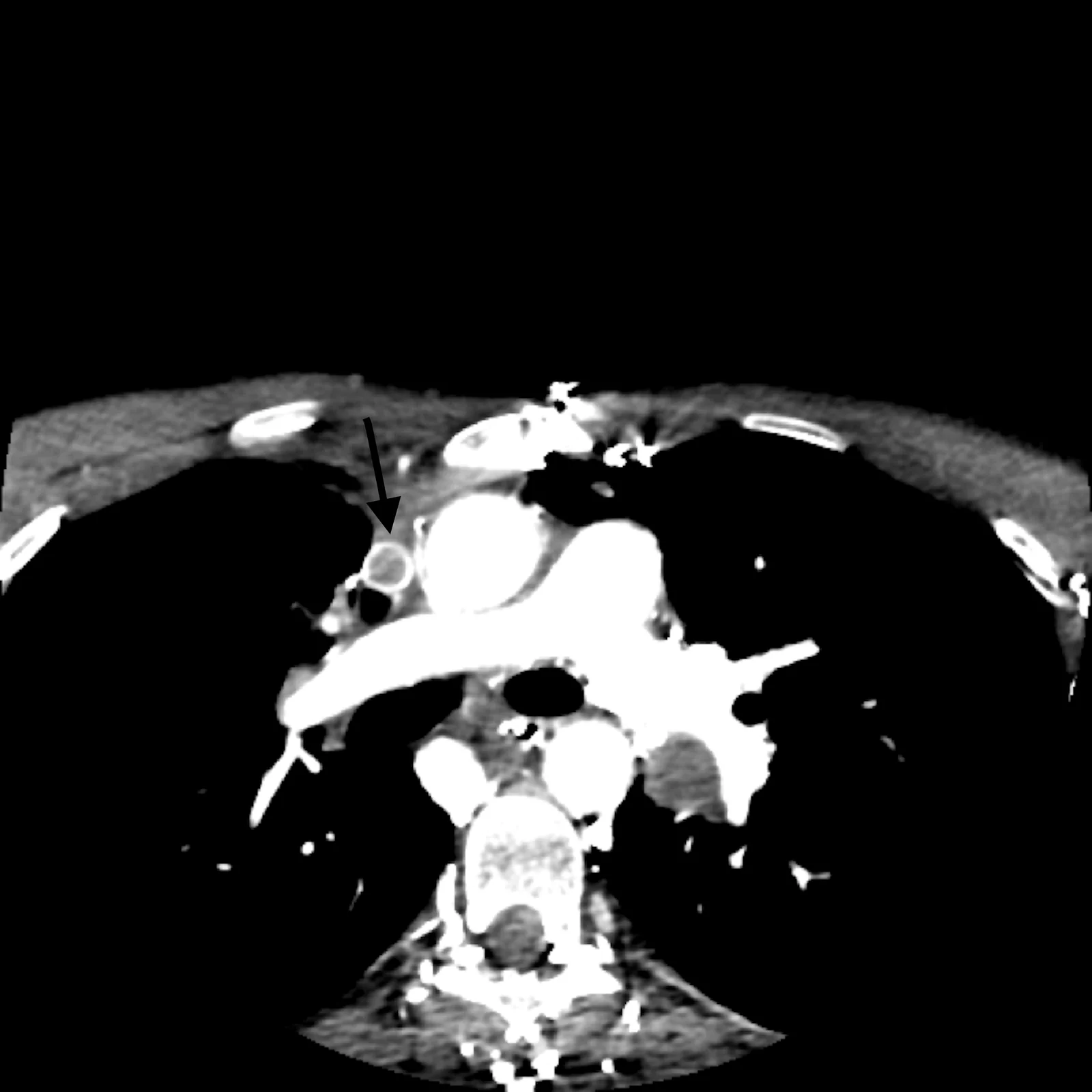
Postoperative contrast-enhanced chest CT axial view showing a patent SVC/PTFE graft SVC: superior vena cava; PTFE: polytetrafluoroethylene

**Figure 8:**
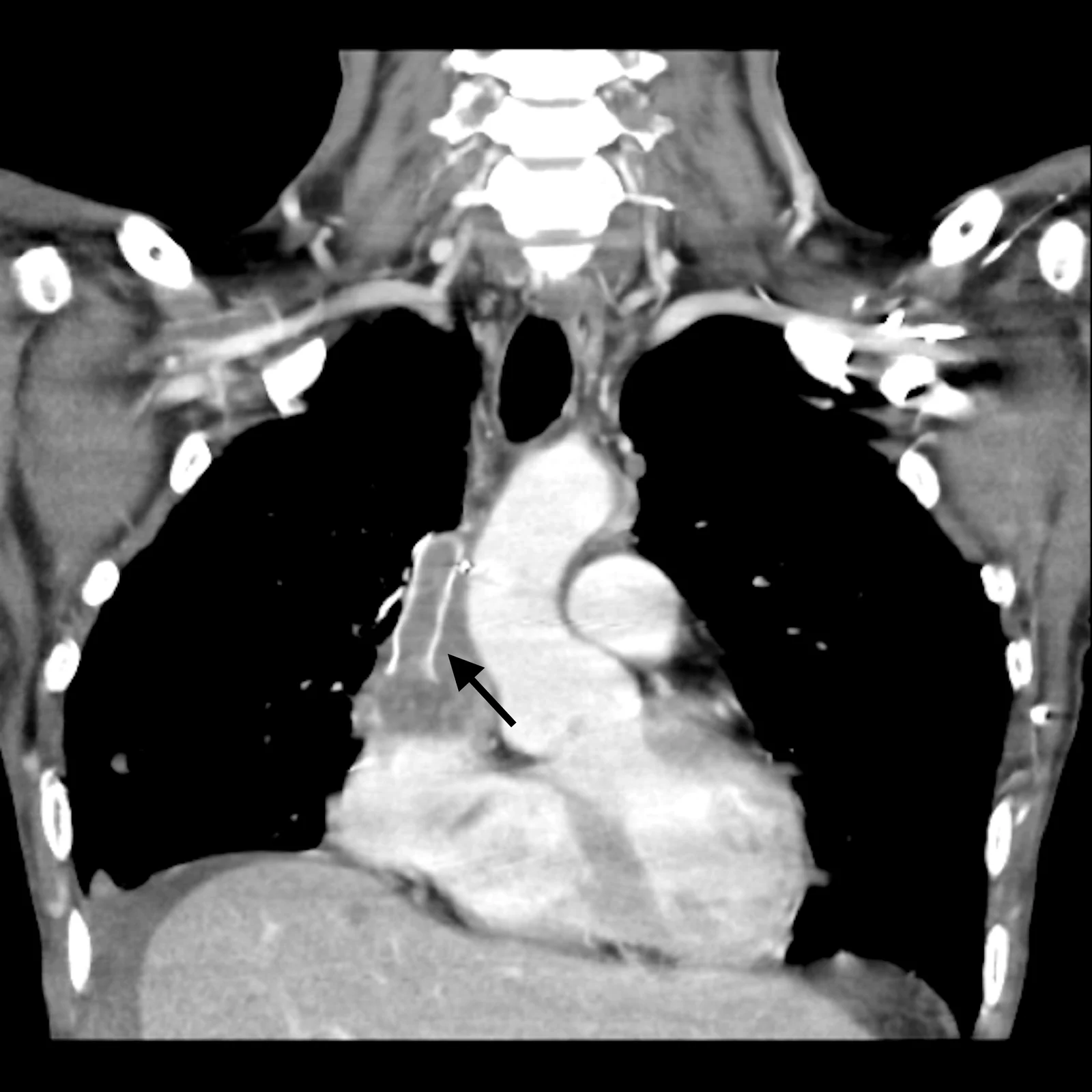
Postoperative contrast-enhanced chest CT sagittal view showing a patent SVC/PTFE graft. The arrow indicates the reconstructed graft segment SVC: superior vena cava; PTFE: polytetrafluoroethylene

The patient tolerated the operation well and was transferred to the Cardiac Surgery Intensive Care Unit (ICU) in stable condition, where he was extubated after two hours. One-month postoperative chest X-ray showed clear lung fields and no recurrent right-sided mediastinal opacity, consistent with absence of the previously seen mass (Figure 6). Postoperative contrast-enhanced CT chest confirmed patency of the reconstructed SVC/PTFE graft on axial and sagittal views (Figures 7-8). The patient had no recurrence of symptoms or other complaints. He was prescribed two additional cycles of adjuvant chemotherapy and continues to be followed in clinic with surveillance imaging.

## Discussion

PMGCTs are rare neoplasms, accounting for less than 5% of all germ cell tumors, with the nonseminomatous subtype comprising approximately 60-70% of mediastinal cases [5]. Unlike their gonadal counterparts, NS-PMGCTs are uniformly malignant, demonstrate aggressive local behavior, and are associated with inferior outcomes. Distant metastases are present in nearly 35% of patients at diagnosis, most commonly involving the lungs, liver, and retroperitoneal lymph nodes [8], [9], underscoring their aggressive nature.

Clinical presentation is typically due to mass effect, with patients commonly reporting chest pain, cough, dyspnea, fever, or weight loss. SVC syndrome has been reported in 20-50% of NS-PMGCT cases, although it is seldom the predominant presenting feature [2]. In contrast, our patient presented with prominent venous distension and respiratory symptoms secondary to complete SVC occlusion, reflecting advanced mediastinal compression at presentation. This pattern places the current case at the severe end of the clinical spectrum described in the literature.

The diagnostic evaluation of NS-PMGCTs remains challenging due to substantial overlap with other anterior mediastinal malignancies, particularly lymphoma, thymoma, thymic carcinoma, and metastatic disease [2,8]. This complexity is further amplified by frequent tumor necrosis and poor differentiation on biopsy specimens. In the present case, initial histopathology demonstrated necro-inflammatory tissue with atypical cells, necessitating an expanded immunohistochemical panel and correlation with serum tumor markers to establish the diagnosis. Positivity for AFP and glypican-3, combined with markedly elevated serum AFP and exclusion of lymphoid and epithelial markers, was critical in confirming NS-PMGCT [10].

Tumor burden at presentation is another defining feature of NS-PMGCTs. Reported median tumor sizes range from 9 cm to 12 cm, while lesions exceeding 15 cm are uncommon but have been described [2], [5]. The mass in our patient measured 15.8 cm, placing it at the upper extreme of reported dimensions and likely accounting for the extensive local complications observed. Pericardial effusion occurs in a minority of cases, whereas progression to cardiac tamponade is rare, reported in approximately 6-7% of patients in larger series [2]. Pleural effusions are similarly infrequent and typically mild [8,9]. Unlike thrombotic SVC obstruction, malignancy-associated SVC syndrome generally requires prompt treatment of the underlying malignancy rather than anticoagulation or endovascular stenting alone. In aggressive lymphomas, early initiation of chemotherapy may rapidly relieve symptoms and improve clinical outcomes. Recognizing SVC syndrome as an oncologic emergency enables timely multidisciplinary evaluation and management.

Current treatment strategies recommend cisplatin-based combination chemotherapy as first-line treatment for NS-PMGCTs [8,9]. Although chemotherapy often results in significant tumor regression, residual masses are common and may represent fibrosis, necrosis, or persistent viable tumor. In the present case, chemotherapy achieved substantial reduction in tumor size but failed to resolve SVC obstruction, illustrating the limitations of systemic therapy in the setting of fixed vascular invasion. Surgical resection is therefore recommended for residual disease following chemotherapy, particularly when viable tumor or ongoing complications are suspected [8]. While most reported surgical interventions involve tumor excision alone, the need for segmental SVC resection with PTFE graft reconstruction under cardiopulmonary bypass, combined with extensive pericardial and pulmonary adhesiolysis, distinguishes this case as surgically complex and rarely reported in the existing literature [9].

## Conclusions

This case highlights the importance of timely diagnosis and coordinated multidisciplinary management in patients with PMGCTs presenting with vascular and cardiac complications. Surgical reconstruction of the SVC using an expanded polytetrafluoroethylene (ePTFE) prosthesis has been reported to provide immediate and durable relief of symptoms associated with SVC obstruction, with low surgical morbidity, including in selected patients with unresectable malignancy. The use of neoadjuvant chemotherapy allowed for tumor response prior to complex resection, while surgical reconstruction of the SVC enabled complete management of the vascular involvement. This case emphasizes that even advanced mediastinal germ cell tumors with invasion or compression of major thoracic structures may be successfully treated through individualized planning involving oncology, thoracic surgery, cardiac surgery, radiology, and critical care teams. Early recognition of compressive symptoms and rapid escalation to multidisciplinary care are essential to improving outcomes in these complex presentations.
